# GMFG Has Potential to Be a Novel Prognostic Marker and Related to Immune Infiltrates in Breast Cancer

**DOI:** 10.3389/fonc.2021.629633

**Published:** 2021-07-23

**Authors:** Yan Yang, Xin He, Qian-Qian Tang, You-Cheng Shao, Wen-Jing Song, Peng-Ju Gong, Yi-Fan Zeng, Si-Rui Huang, Jiang-Yao Zhou, Hui-Fang Wan, Lei Wei, Jing-Wei Zhang

**Affiliations:** ^1^ Department of Breast and Thyroid Surgery, Zhongnan Hospital, Wuhan University, Wuhan, China; ^2^ Department of Pathology and Pathophysiology, School of Basic Medicine, Wuhan University, Wuhan, China

**Keywords:** GMFG, prognosis, tumor-infiltrating immune cells, WGCNA, CIBERSORT, breast cancer

## Abstract

A growing amount of evidence has indicated immune genes perform a crucial position in the development and progression of breast cancer microenvironment. The purpose of our study was to identify immunogenic prognostic marker and explore potential regulatory mechanisms for breast cancer. We identified the genes related to ImmuneScore using ESTIMATE algorithm and WGCNA analysis, and we identified the differentially expressed gene (DEGs). Then, Glia maturation factor γ (GMFG) was determined as a predictive factor by intersecting immune-related genes with DEGs and survival analysis. We found the expression of GMFG was lower in breast cancer tissues compared with normal breast tissues, which was further verified by immunohistochemical (IHC). Moreover, the decreased expression of GMFG was significantly related to the poor prognosis. Besides, the expression of GMFG was related to the age, ER status, PR status, HER2 status and tumor size, which further suggested that the expression of GMFG was correlated with the subtype and the growth of tumor. The univariate and multivariate Cox regression analyses revealed that age, stage, the expression level of GMFG and radiotherapy were independent factors for predicting the prognosis of breast cancer patients. Subsequently, a prognostic model to predict the 3-year, 5-year and 10-year overall survival rate was developed based on the above four variables, and visualized as a nomogram. The values of area under the curve of the nomogram at 3-year, 5-year and 10-year were 0.897, 0.873 and 0.922, respectively, which was higher than stage in prognostic accuracy. In addition, we also found that GMFG expression level was correlated with sensitivity of some breast cancer chemotherapy drugs. Furthermore, the results of GSEA indicated immune-related pathways were mainly enriched in GMFG-high-expression group. CIBERSORT analysis for the proportion of tumor-infiltrating immune cells (TIICs) suggested that expression of GMFG was positively association with multiple kinds T-cell in BC. Among them, CD8+ T cells had the strongest correlation with GMFG expression, which revealed that GMFG might has an antitumor effect by increasing the infiltration of CD8+ T cells in breast cancer. Accordingly, GMFG has the potential to become a novel immune biomarker for the diagnosis and treatment of breast cancer.

## Introduction

Breast cancer was the most commonly diagnosed cancer among women in the majority of countries and the most common malignant tumor that seriously threaten health of women. For women, it is predicted that newly diagnosed breast cancer alone will accounts for 30% of female cancers in 2020. Moreover, the incidence of breast cancer has continued to rise in the past decade. Although great advances have been made in the earlier diagnosis and therapy over two decades, the mortality rate of breast cancer has not dropped significantly. Breast cancer is still the main reason of cancer deaths in more than 100 countries and in women aged 20 to 59 years (1–3). Therefore, it is urgent to search effective markers for early diagnosis, prognosis prediction and treatment of breast cancer.

The tumor microenvironment(TME) significantly affects treatment response and clinical prognosis of cancer patients. Tumor microenvironment was a complex environment for tumor cells to survive, which was composed of a series of cytokines, infiltrating immune cells and tumor cells (4, 5). Increasing evidence demonstrated that tumor-infiltrating immune cells (TIICs) in the TME can promote tumor cell migration and invasion, pro-angiogenesis, drug resistance and evading immune surveillance (6–8). T reg cells inhibit tumor-associated antigen presentation, and also interfere with the function of cytotoxic T cell by suppressing release of cytolytic granule. In some tumors, including breast cancer, increased T reg cells was related to poor overall survival (OS) (9). However, different types of TIICs may play contradictory roles in cancer. For example, evidence supports a positive role for tumor-infiltrating B cells in antitumor immunity (10). In addition to lymphocytes, tumors usually include tumor-associated macrophages (TAM) which was reported to be correlated with shorter disease-free survival (DFS) and OS in breast cancer (11, 12). Some studies shown that the degree infiltration of macrophage in tumor tissue was also related to grade, tumor size, subtype and receptor status of breast cancer (13, 14). Moreover, targeting TAMs can reduce tumor cell invasion, metastasis, angiogenesis, and enhance the anti-tumor activity of chemotherapy (15). These results indicated that the TIICs might play very important role in the progression of breast cancer.

In the present study, we screened out the immune-related gene glia maturation factor γ (GMFG) that was down-regulated in breast cancer through the WGCNA analysis and differential analysis of cancer tissues and adjacent tissues. Then, we analyzed the correlation between expression of GMFG and clinical characteristics, survival and the sensitivity of chemotherapy drugs. In order to explore the function of GMFG in tumor immunity, we analyzed the immune-related signaling pathways using GSEA, and the correlation and difference of TIICs infiltration between breast cancer (BC) tumor samples with low or high GMFG expression group. Furthermore, we also investigated the co-expressed genes of GMFG. We hope this study will contribute to prognostic monitoring and treatment strategies for BC patients.

## Materials and Methods

### Raw Data

We downloaded breast cancer dataset, namely GSE42568, from the Gene Expression Omnibus database ((https://www.ncbi.nlm.nih.gov/geo/query/acc.cgi?acc=GSE42568). GSE42568 contained 104 breast cancer specimens and 17 normal breast tissues specimens. Transcriptome fragments per kilobase million (FPKM) data and clinicopathology data of breast cancer were downloaded from The Cancer Genome Atlas (TCGA, https://portal.gdc.cancer.gov/). After excluding patients with incomplete clinical information, we enrolled 743 breast cancer samples. In addition, mRNA expression data and clinical information of another 1077 breast tumor samples were obtained from METABRIC website (https://ega-archive.org/access/data-access).

### Identification of DEGs

The samples of GSE42568 dataset were processed and standardized with R and log2 converted. We used the “limma” package in R to identify DEGs. The cut-off criteria were log2 fold-change (logFC) >1 and *p <*0.05.

### Estimation of Stromal and Immune Scores

We used “estimate” package in R to calculate immune cell infiltration level (Immunescore), stromal content (Stromalscore), and the sum of both (Estimatescore) for each sample from TCGA database.

### Construction of Co-Expression Network

Expression of genes with highest 25% of variance (4895 genes) were selected for further co-expression network analysis using the “WGCNA” package. Firstly, we built an adjacency matrix to depict the degree of correlation between the nodes and chose β = 4 (scale free R^2^ = 0.89) as soft-threshold to ensure the construction of scale-free network in our study. Then, the adjacency matrix was transformed into a topological overlap matrix (TOM), which was a method called the network connectivity of a gene to measure the sum of adjacent degree between the gene and all other genes. Subsequently, we performed average linkage hierarchical clustering according to the TOM-based dissimilarity measurements with a minimum size (gene group) of 50 for the genes dendrogram, and classified genes with similar expression profiles into the same gene modules.

### Identifying Module With the Highest Correlation With ImmuneScore

We calculated the correlation between module eigengenes and immune-stromal score to determine the significance of modules by Pearson test. The module with the highest correlation coefficient with ImmuneScore was selected and defined as a hub module.

### Functional Enrichment Analysis and PPI Network Construction

The DAVID database (https://david.ncifcrf.gov/) was used to perform gene ontology (GO) enrichment analysis and The Kyoto Encyclopedia of Genes and Genomes (KEGG) enrichment analysis. Then the enrichment results were visualized by the “GOplot” R package and the “ggplot2” R package. The protein–protein interactive (PPI) network was constructed by the STRING database (http://string-db.org).

### Survival Analysis

Based on the median expression of mRNA, we divided the BC patients from TCGA database and METABRIC database into a high expression group and a low expression group. We used the “survival” R package to draw the Kaplan-Meier survival curves of the samples, and performed the log-rank test for comparison. *p*< 0.05 indicated that the difference was statistically significant. Besides, we performed univariate and multivariate Cox regression analysis to identify factors correlated with OS. The prognostic value of GMFG in breast cancer samples was further assessed by Kaplan-Meier Plotter (http://kmplot.com/analysis/index.php?p=service&cancer=breast).

### TIMER Database Analysis and Prediction of Co-Expression Genes

The “Differential Expression” module of TIMER database (https://cistrome.shinyapps.io/timer/) was used to analyze the expression level of GMFG in cancer tissues and adjacent tissues. LinkedOmics database (http://www.linkedomics.org/login.php) was used to obtain the co-expression genes associated with GMFG.

### Development a Prognostic Nomogram

Based on independent prognostic parameters, Cox regression model was used to establish a prognostic nomogram for predicting 3-year, 5-year and 10-year OS in BC patients. We used receiver operating characteristic curves (ROC) to evaluate the predictive ability of the prognostic nomogram.

### Chemotherapy Sensitivity Analysis

We accessed the NCI-60 database through the CellMiner website (https://discover.nci.nih.gov/cellminer), which contained 60 different cancer cell lines from 9 different types of tumors. Pearson correlation analysis was performed to investigate the association between the GMFG expression and sensitivity of breast cancer chemotherapy drugs.

### Gene Set Enrichment Analysis (GSEA)

We explored the potential functions of GMFG by gene set enrichment analysis (GSEA) using BC samples from TCGA database. Briefly, according to the median expression levels of GMFG, 743 BC patients were grouped into GMFG-high-expression group and GMFG-low-expression group. Besides, we selected “c2.cp.kegg.v7.0.symbols.gmt” as the reference gene set. We considered the KEGG signaling pathway with *p*<0.05 were significantly enriched.

### Correlation and Difference Analysis of TIICs Profile

The CIBERSORT algorithm (https://cibersort.stanford.edu/) was used to analyze the proportion of 22 kinds of the TIICs in all BC tumor samples from the TCGA database. We still analyzed the relation between the expression level of GMFG and the ratio of TIICs and drawn a scatter plot using the “ggpubr” R package, and used the Pearson coefficient to test the correlation. In addition, we drawn violin plots using “vioplot” R package to display the difference in TIILs between GMFG-high-expression group and GMFG-low-expression group, and the significance tested by Wilcoxon rank sum.

### Verification of the Protein Expression of GMFG by Immunohistochemistry (IHC)

The breast cancer tissue microarray (F048Br01) was purchase from Zhong Ke Guang Huang Biotech (http://bioaitech.com), which contained 24 paired breast tumor tissues and adjacent non-tumorous tissue samples. IHC was performed on the tissue microarray using the UltraSensitive™S-P Methods. Briefly, the tissue samples were dewaxed and treated with methanol containing 3% hydrogen peroxide to inactivate the endogenous peroxidase. After that, the tissue samples were incubated with primary antibody for GMFG overnight at 4°C, followed by incubated with secondary antibody (HRP polymer) for 30 minutes. Then, diaminobenzoquinone (DAB) was used to develop. Finally, hematoxylin was applied to counterstain the microarrays. Staining index was equal to the product of staining intensity score and the score of positive tumor cells. The intensity of staining was scored according to: 0 (no staining, -); 1 (weak staining, light yellow,+); 2 (moderate staining, yellow brown,++); 3 (strong staining, brown,+++). Tumor cells proportion was scored as follows: 0 (no positive tumor cells); 1 (<10% positive tumor cells); 2 (10%-25% positive tumor cells); 3 (26%-49% positive tumor cells); 4 (≥50% positive tumor cells). The primary antibody used in our work was Anti-GMFG(1:500, Proteintech).

### Statistical Analysis

We performed statistical analysis by R software v3.6.0 (https://www.r-project.org/). Chi-squared test or Fisher’s exact test were used to analyze categorical variables, and Student’s t test to analyze continuous variables. Survival rate was assessed using Kaplan-Meier curves and the log-rank test, and univariate and multivariate Cox regression were used to analyze the independent parameters associated with the OS. We used Wilcoxon test to analyze the expression difference between breast cancer tissues and tumor-adjacent normal tissues and difference of TIICs between GMFG-high-expression group and GMFG-low-expression group. The correlation between two variables was measured by Pearson coefficient of correlation. Two-tailed value of *p*< 0.05 was considered statistically significant.

## Results

### Identification of DEGs

**Figure 1:**
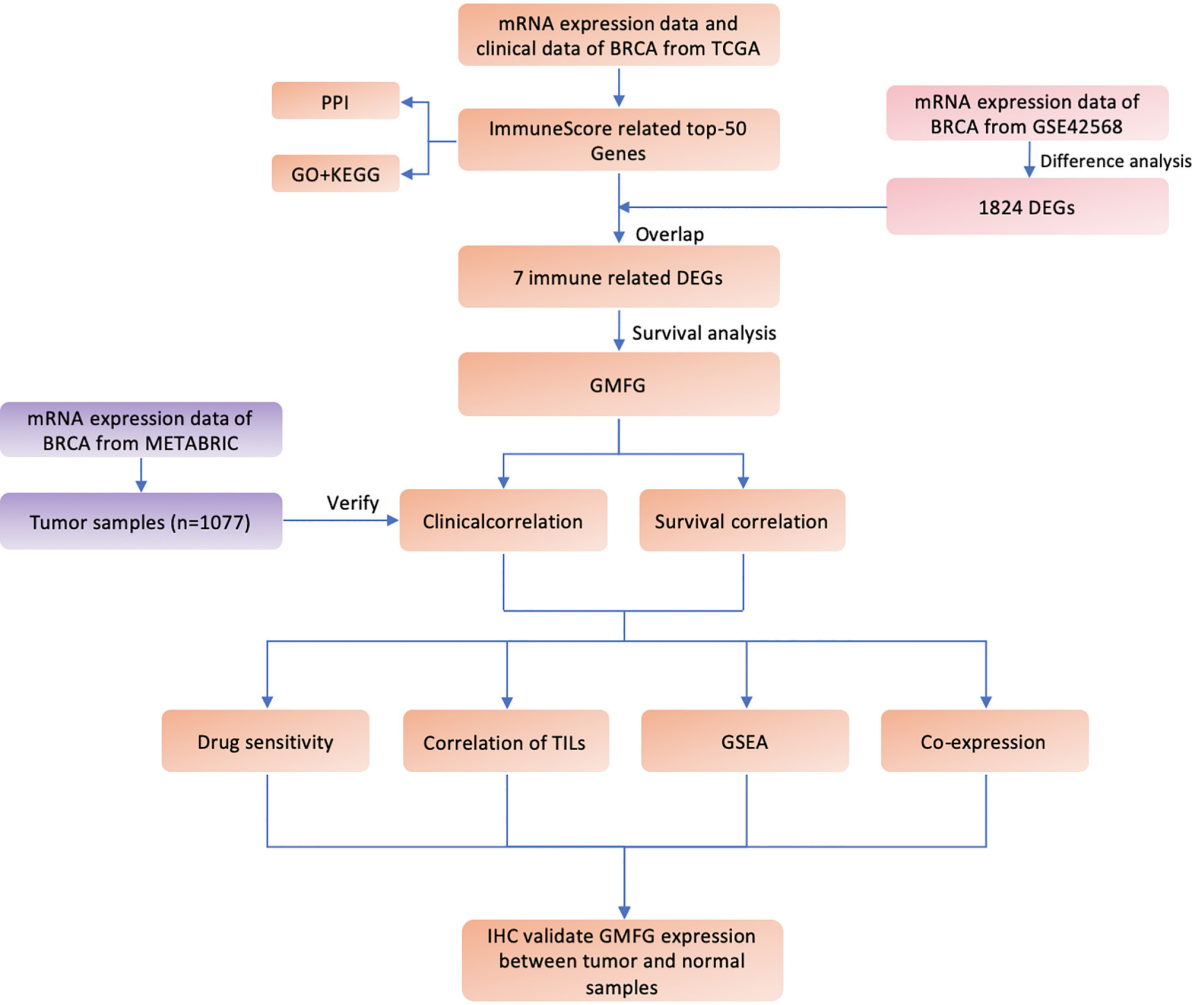
Flow diagram of this study.

**Figure 2:**
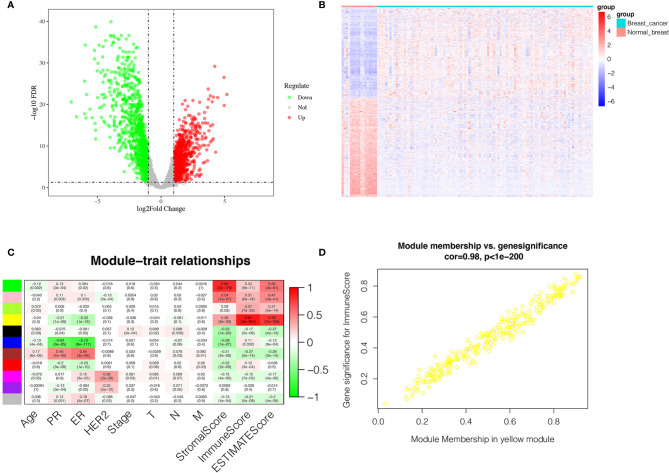
Identification of DEGs and IRGs. **(A)** Volcano diagram of DEGs which include 874 up-regulated genes and 957 down-regulated genes. **(B)** The heatmap of DEGs expression profile in breast cancer group (n = 104) and normal breast tissues group (n = 17). **(C)** Correlation between the gene module and clinical traits including Stromalscore, Immunescore and Estimatescore. Each row represents eigengene of module, column represents trait. The former numbers in each box was the correlation coefficient and the numbers in brackets indicate the p-value for the correlation. **(D)** A scatterplot of Gene Significance (GS) for Immunescore vs. Module Membership (MM) in the yellow module.

**Figure 1** shown the workflow of our study. Comparing breast cancer tissues and normal breast tissues in the GSE42568 processed and standardized with R and log2 converted, 1831 DEGs were identified, with 874 up-regulated genes and 957 down-regulated genes in the breast cancer (FDR < 0.05 and |logFC| > 1). The volcano plot and the heatmap for the up- and down-regulated genes was displayed in **Figures 2A, B**, respectively.

### Identification of Immune-Related Genes (IRGs)

The transcriptome and clinical data of 743 breast cancer cases were downloaded from the TCGA database. Subsequently, the stromal score and immune score were calculated using ESTIMATE algorithm to estimate the tumor purity for each BC sample, and the fractions of Immunescore, Stromalscore, and Estimatescore were used as trait data of WGCNA. According to the analysis of WGCNA, eleven modules were identified and the correlation between these modules and clinical traits was analyzed (**Figure 2C**). The yellow module was highly related to ImmuneScore, and was selected as a hub module and the top 50 intramodule connectivity genes were screened as immune-related genes (IRGs). Then, we plot a scatterplot of Gene Significance *vs*. Module Membership in the yellow module (**Figure 2D**). Subsequently, we constructed a PPI network to further explore the interaction between the IRGs using STRING database. As shown in **Supplementary Figure 1A**, 50 nodes has 386 edges in the network, with average node degree of 15.4 and average local clustering coefficient of 0.654, which further verify the strong interaction between the IRGs. Moreover, in order to further explore the functions of IRGs, we performed GO enrichment analysis and KEGG enrichment analysis using DAVID database. The KEGG pathway enrichment analysis results revealed that the IRGs was almost enriched in the immune-related pathway and function (**Supplementary Figure 1B**), such as primary immunodeficiency, T-cell receptor signaling pathway, cell adhesion molecules (CAMs), hematopoietic cell lineage and cytokine-cytokine receptor interaction. In addition, GO enrichment analysis (**Supplementary Figures 1C–F**) also displayed the enrichment of immune-related GO terms, including 3 categories: (1) biological process (BP): positive regulation of T cell proliferation, immune response, leukocyte migration and regulation of immune response; (2) cellular component (CC): immunological synapse and T cell receptor complex; and (3) molecular function (MF): protein kinase binding, MHC class II protein binding and transmembrane signaling receptor activity.

### Identification of Immune-Related DEGs

**Figure 3:**
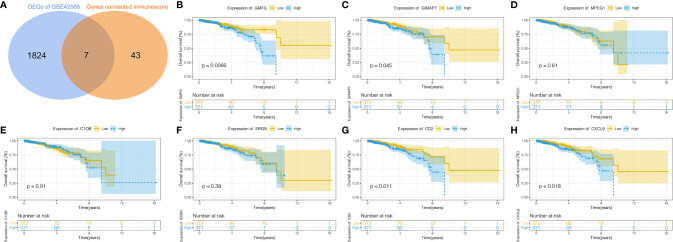
Determination of Immunescore-related DEGs and their correlation with survival in breast cancer. **(A)** Overlapping of Immunescore-related genes and DEGs result in seven intersecting genes and these genes were selected as hub genes. **(B–H)** Kaplan–Meier survival curve of seven hub genes.

The intersection analysis between the IRGs and the DEGs was carried out, and seven genes(MPEG1, GMFG, SRGN, CXCL9, GIMAP7, CD2 and C1QB) were overlapping from the above analyses and were selected as hub genes (**Figure 3A**). Based on the median expression of hub genes, we grouped BC patients into high-expression group and low-expression group. We used Kaplan-Meier survival analysis to analyze the correlation between the each hub genes with OS (**Figures 3B–H**). The results indicated that CD2, CXCL9, GIMAP7 and GMFG were associated with the OS rate of BC patients (*p*< 0.05), but not with the other three genes. Next, the gene with a p-value of less than 0.01 in survival analysis was selected to further analyze, that is GMFG. The result of survival analysis indicated that the survival time of BC patients in GMFG-high-expression group was longer than GMFG-low-expression group.

### Validation of the Expression of GMFG in the TCGA Cohort

**Figure 4:**
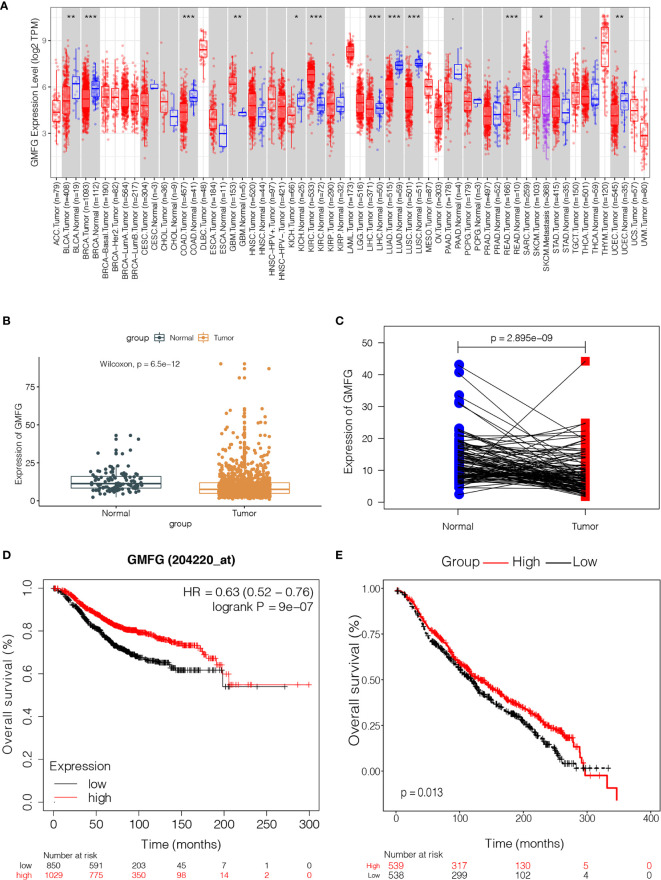
The expression level of GMFG in breast cancer and its correlation with survival in breast cancer. **(A)** The differential expression of GMFG in multiple tumor types were analyzed by TIMER online platform (*p < 0.05, **p < 0.01, ***p < 0.001). **(B)** Differentiated expression of GMFG in the normal breast tissues and breast cancer tissues, which was assessed by the Wilcoxon test (***p < 0.001). **(C)** Paired differentiation analysis was used to analyze GMFG expression in tumor-adjacent normal tissues and tumor tissues from the same patient, which was also assessed by the Wilcoxon test (***p < 0.001). **(D)** Prognostic significances of GMFG expression in breast cancer patients based on the Kaplan-Meier Plotter database. **(E)** Prognostic significances of GMFG expression in breast cancer patients based on the METABRIC database.

In order to verify the difference in expression of GMFG between tumors and normal tissues, we used TIMER database to analyze the expression of GMFG in tumor and normal tissues of multiple cancer types. The results indicated that expression of GMFG was lower in bladder urothelial carcinoma, breast cancer, colon adenocarcinoma, kidney chromophobe, lung adenocarcinoma, lung squamous cell carcinoma, liver hepatocellular carcinoma, pancreatic adenocarcinoma, rectum adenocarcinoma and uterine corpus endometrial carcinoma than in normal tissues. But, expression of GMFG in glioblastoma multiforme and kidney renal clear cell carcinoma was higher than expression in normal tissues (**Figure 4A**). The expression of GMFG was further tested in TCGA database. Consistent with the result of TIMER, the GMFG expression in breast cancer were downregulated compared within normal breast tissue (**Figure 4B**), and the pairing analysis between tumor-adjacent normal tissues and breast cancer tissues from the same patient also confirmed similar result (**Figure 4C**).

### Validation of the Correlation Between GMFG Expression Level and OS and Clinical Features

**Figure 5:**
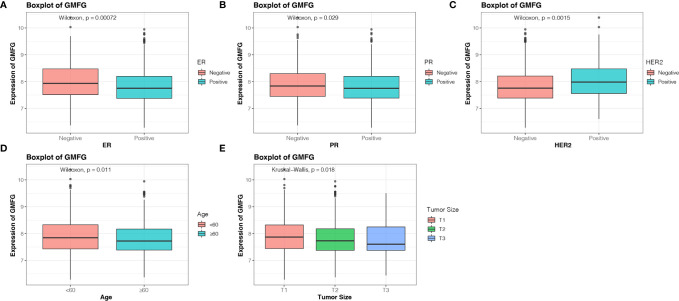
Correlation analysis between the expression of GMFG with clinical and pathological features. **(A)** Estrogen receptor (ER) status. **(B)** Progesterone receptor (PR) status. **(C)** Human epidermal growth factor receptor 2 (HER2) status. **(D)** Age. **(E)** Tumor size (T1: ≤ 2cm, T2: > 2cm and ≤ 5cm, T3: > 5cm).

The Kaplan-Meier Plotter was used to further verify the correlation between the expression level of GMFG and the survival. The Kaplan-Meier curve confirmed that the low expression of GMFG was negatively correlated with the OS (**Figure 4D**). In addition, we download expression data of GMFG from METABRIC database, and excluded cases with incomplete clinical data of breast cancer patients from METABRIC database. Finally, 1077 cases were included. We divided the BC patients into high and low expression group based on the median expression of GMFG and then performed survival analysis. The result showed in **Figure 4E**, which also indicated that the low expression level of GMFG was associated with poor prognosis in breast cancer (*p*=0.013). Subsequently, we analyzed the association of GMFG expression with clinical and pathological features. As shown in **Figure 5**, The results showed that the expression of GMFG was significantly associated estrogen receptor (ER) status (*p*<0.001), progesterone receptor (PR) status (*p*=0.029) and human epidermal growth factor receptor 2 (HER2) status (*p*=0.002). And the expression of GMFG in breast cancer patients older than 60 years old is significantly higher than that in breast cancer patients younger than 60 years old (*p*=0.011). Besides, the expression level of GMFG showed the significant negative correlation with tumor size (*p*=0.018).

### Prognostic Potential of GMFG in Breast Cancer

**Figure 6:**
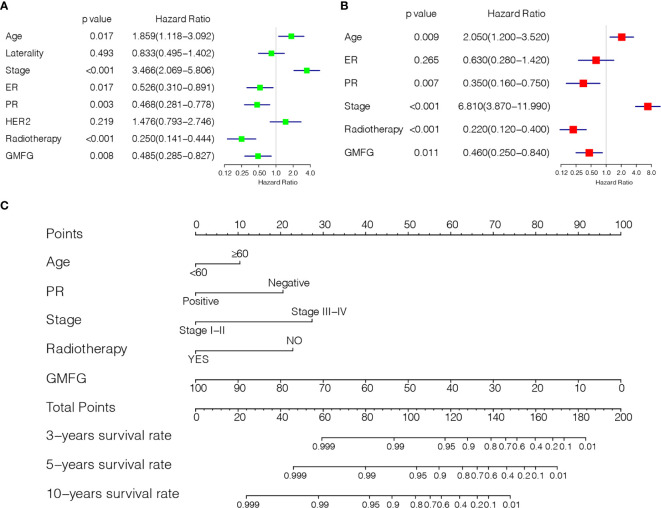
Establishment of Prediction model. **(A)** Forest plot of univariable Cox regression analyses. **(B)** Forest plot of multivariable Cox regression analyses. **(C)** Nomogram combining the GMFG expression with clinicopathological features.

**Figure 7:**
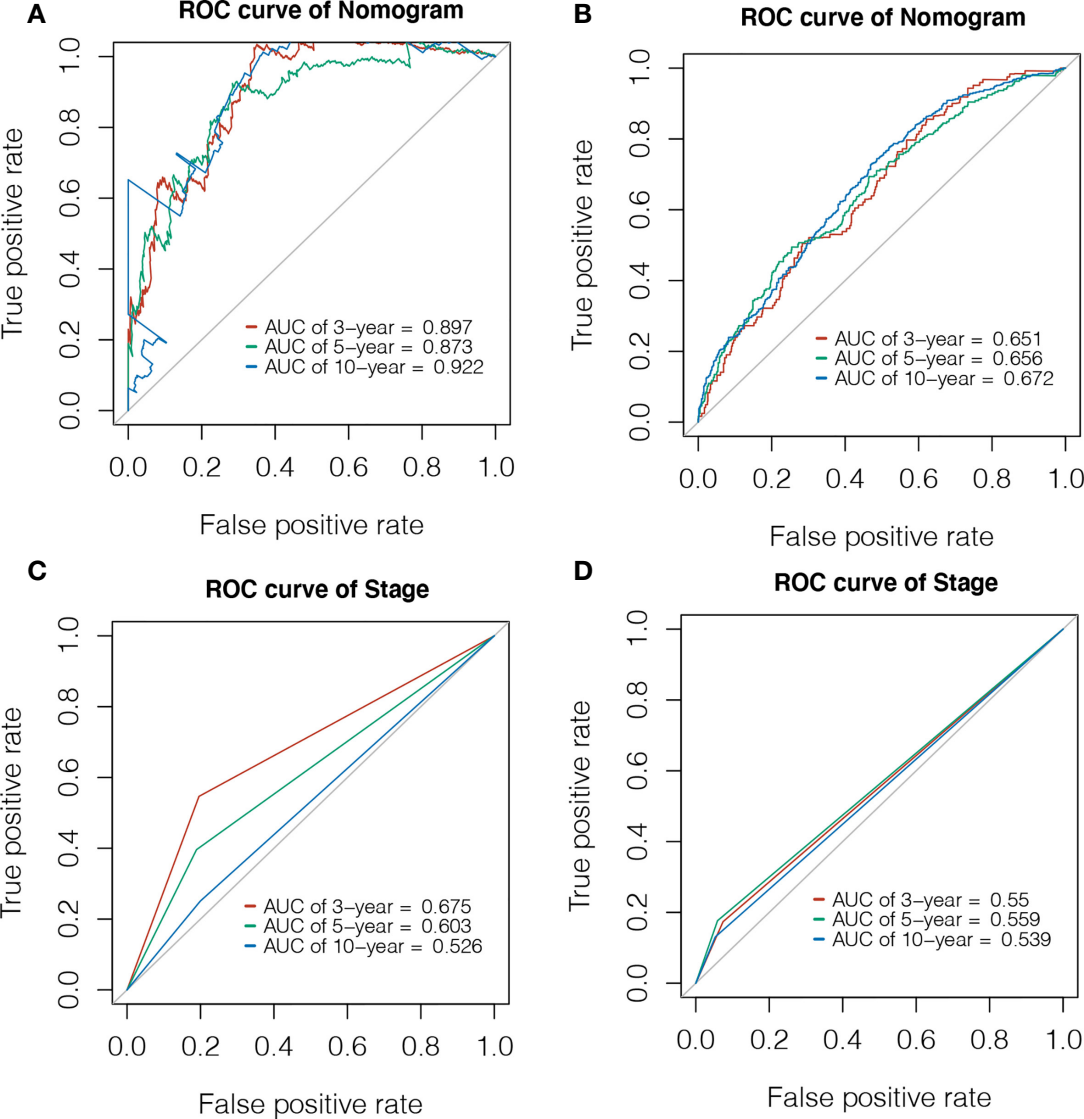
Evaluation of Prediction model. **(A)** Time-dependent ROC curve was used to evaluate the prognostic accuracy of nomogram in 3-year, 5-year and 10-year based on the TCGA database. **(B)** Time-dependent ROC curve was used to validate the prognostic accuracy of nomogram in 3-year, 5-year and 10-year based on the METABRIC database. **(C)** Time-dependent ROC curve was used to evaluate the prognostic accuracy of stage in 3-year, 5-year and 10-year based on the TCGA database. **(D)** Time-dependent ROC curve was used to validate the prognostic accuracy of stage in 3-year, 5-year and 10-year based on the METABRIC database.

Using the expression of GMFG and clinical features (age, laterality, stage, ER status, PR status, HER2 status and Radiotherapy) as covariates, univariate and multivariate Cox regression analysis were performed to determine independent prognostic factors (**Figures 6A, B**). The results indicated age, stage, GMFG expression and radiotherapy were the independent prognostic factors. Subsequently, we established a nomogram, a visualization of the prediction model integrating the GMFG expression levels and three clinical factors (age, stage and radiotherapy), to predict 3-year, 5-year and 10-year survival rate of BC patients (**Figure 6C**). The results of the ROC curve analysis showed that the receiver operating characteristic curves (AUC) values of nomogram were 0.897, 0.873 and 0.922 at 3-year, 5-year and 10-year, respectively (**Figure 7A**). Then, METABRIC database was used to verify the prediction performance of the nomogram, and the AUC values were 0.651, 0.656 and 0.672, respectively (**Figure 7B**). These results suggested the nomogram had good predictive accuracy of prognosis. Besides, we compared the prediction performance of the nomogram and stage, and the results indicated that the prediction performance of the nomogram was better than that of the stage wherever in TCGA dataset or in METABRIC dataset (**Figures 7C, D**).

### GMFG Expression and Cancer Cell Sensitivity to Chemotherapy Drugs

**Figure 8:**
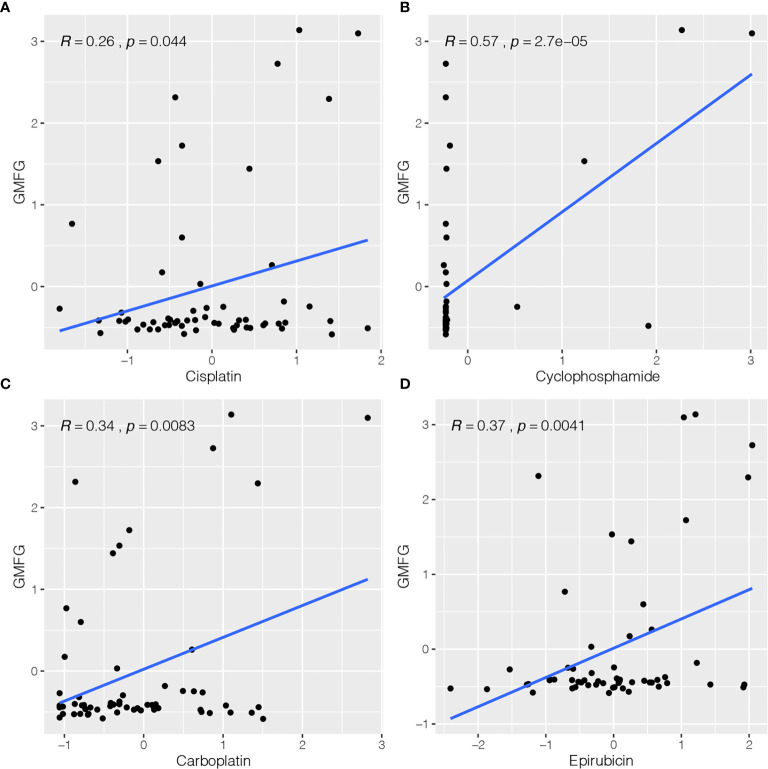
Correlation between expression of GMFG and chemotherapy drugs sensitivity of cancer cells. **(A)** Scatter plot of relationship between GMFG expression and cisplatin sensitivity. **(B)** Scatter plot of relationship between GMFG expression and cyclophosphamide sensitivity. **(C)** Scatter plot of relationship between GMFG expression and carboplatin sensitivity. **(D)** Scatter plot of relationship between GMFG expression and epirubicin sensitivity.

The expression of GMFG was investigated in the NCI-60 cell line, and we analyzed the correlation between its expression level and drug sensitivity. The results suggested that GMFG expression level was correlative to sensitivity of some breast cancer chemotherapy drugs (**Figure 8**). For example, increased expression of GMFG was associated with increased drug sensitivity of cancer cells to cisplatin (r=0.26, *p*=0.044), cyclophosphamide (r=0.57, *p*<0.001), carboplatin (r=0.34, *p*=0.008) and epirubicin (r=0.37, *p*=0.004).

### Expression of GMFG Was Related to Immune Response

**Figure 9:**
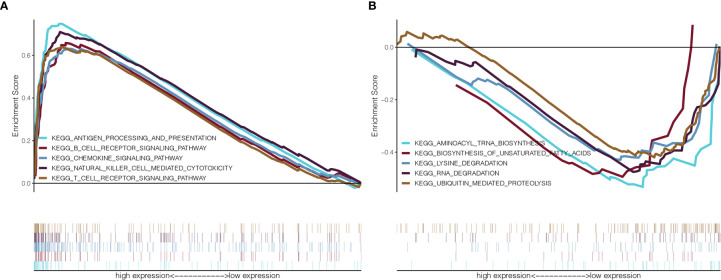
GSEA for GMFG-high-expression group and GMFG-low-expression group. **(A)** GSEA showed that expression of GMFG in breast cancer was positively associated with gene set in KEGG related to immune. **(B)** The expression of GMFG was negatively associated with gene set in KEGG related to metabolic.

To identify the potential involvement of biological pathways and processes in BC patients with GMFG expression, we implemented GSEA in the GMFG-high-expression group and the GMFG-low-expression group. As shown in **Figure 9**, high expression of GMFG was positively enriched in pathways related to immune response, including T cell receptor signaling pathway, B cell receptor signaling pathway, antigen processing and presentation and NK cell mediated cytotoxicity, and was negatively enriched in pathways related to metabolic, such as unsaturated fatty acid biosynthesis, lysine degradation, RNA degradation and ubiquitin-mediated proteolysis. These results suggested that GMFG expression played an important role in immune reaction.

### Expression of GMFG Was Related to TIICs in Breast Cancer

**Figure 10:**
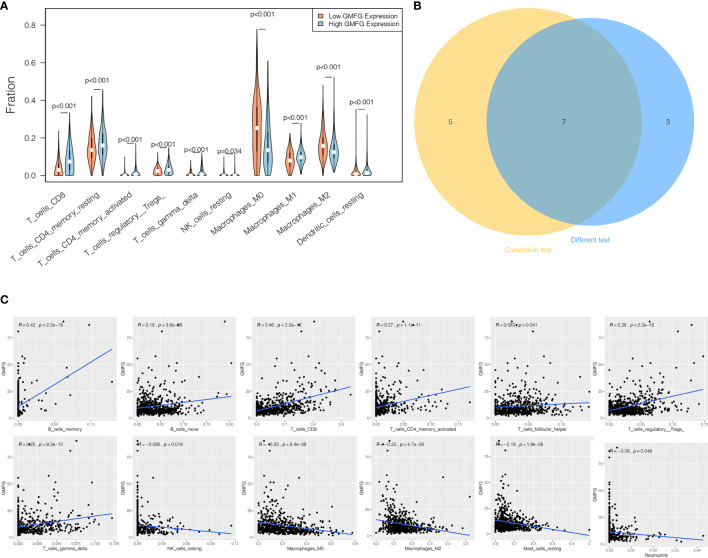
Correlation between proportion of TIICs and expression of GMFG. **(A) **The violin chart displayed 10 kinds of immune cells with significant differences between GMFG-low-expression and GMFG-high-expression groups grouped by the median expression of GMFG. **(B)** The Venn diagram shown the 7 TIICs related to the GMFG expression, which are determined by the correlation and difference tests shown by the scatter diagram and the violin diagram, respectively. **(C)** The scatter diagram displayed the correlation between the expression of GMFG and the proportions of 12 TIICs (* p <* 0.05).

We analyzed the proportion of TIICs subsets using CIBERSORT algorithm to verify the relationship between GMFG expression with TIICs, and drawn immune cell profiles of BC cases and correlation heatmap of 22 kinds TIICs (**Supplementary Figures 2A, B**). The results of the correlation and difference analysis suggested that there were a total of seven TIICs related to the expression of GMFG. Among them, four types of TIICs were positively associated with expression of GMFG, including CD8 + T cells, activated CD4 + memory T cells, γ-δ T cells and regulatory T cells (Tregs). However, three types of TIICs were negatively associated with expression of GMFG, including resting NK cells, macrophage M2 and macrophage M0. These results also demonstrated that the expression of GMFG effected the immune response in breast cancer (**Figure 10**).

### Co-Expressed Genes of GMFG

**Figure 11:**
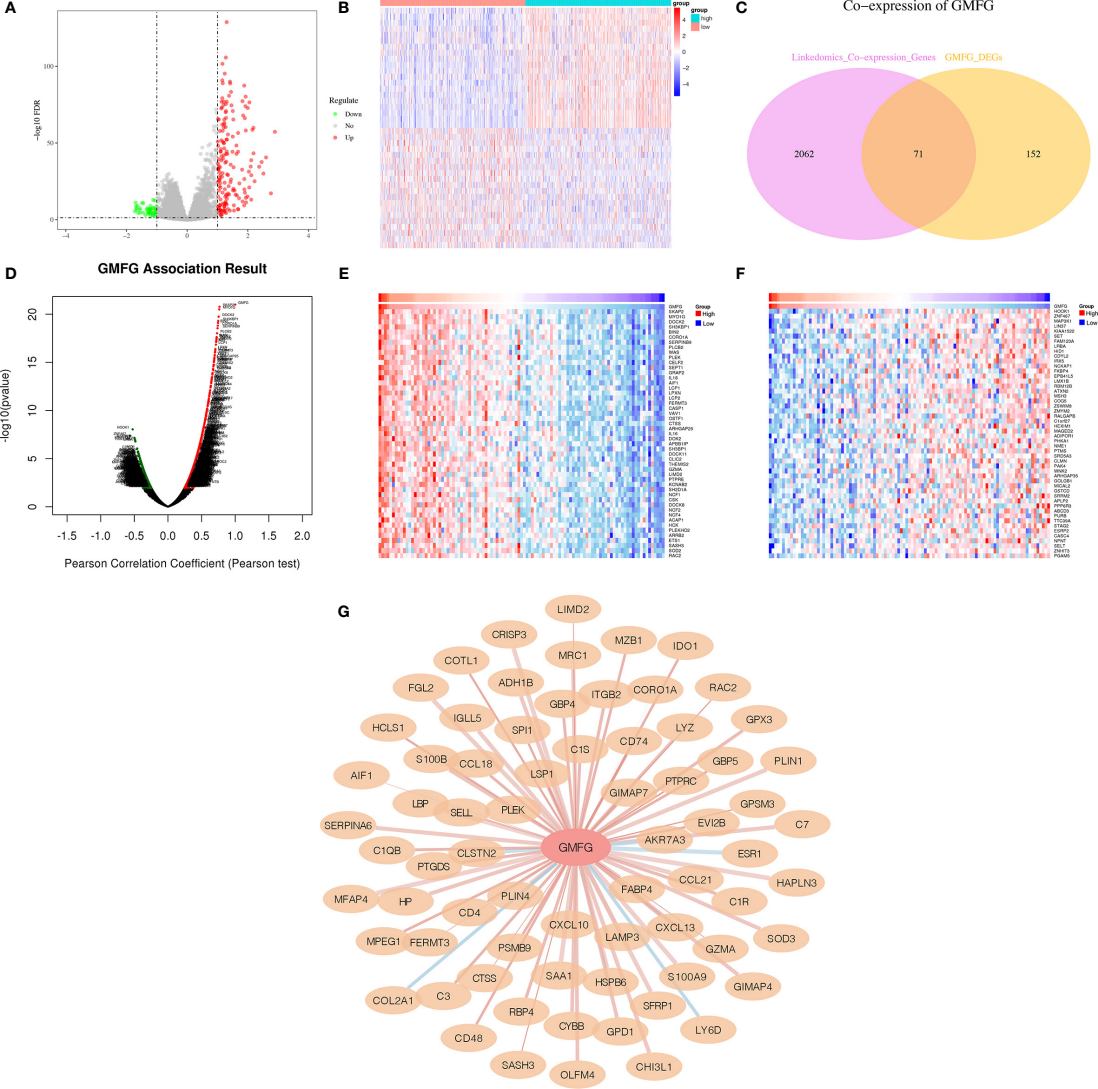
Co-expressed genes of GMFG. **(A)** The volcano plots of differentially expressed genes of is based on differences in expression levels of GMFG. **(B)** The heatmap plots of top 20 up-regulated and down-regulated genes is based on differences in expression levels of GMFG. **(C)** Venn plot displayed 71 genes correlated with GMFG expression codetermined by GMFG-coexpressed genes from LinkedOmics and differential expression genes based on GMFG expression. **(D)** Pearson test in LinkedOmics was used to analyze co-expressed genes of GMFG in breast cancer. **(E)** The top 50 genes in LinkedOmics positively related to expression of GMFG in breast cancer. **(F)** The top 50 genes in LinkedOmics negatively related to expression of GMFG in breast cancer. **(G)** The network diagram shows the interaction between GMFG and its co-expressed genes. The red edges indicated a positive correlation with GMFG, and the blue edges indicated a negative correlation with GMFG. The width of the edge represented the strength of the correlation.

When comparing GMFG-high-expression and GMFG-low-expression, 223 genes were differentially expressed based on the selection criteria of differential genes |log2FC| > 1, FDR < 0.05, including 48 down-regulated and 175 up-regulated differential genes. The volcano plot displayed the up-regulated and down-regulated differential genes in **Figure 11A** and the heatmap displayed the expression of the top 20 up-regulated and down-regulated genes (**Figure 11B**). Moreover, we analyzed the co-expressed genes of GMFG in BC patients using the LinkedOmics database. As displayed in **Figure 11D**, there were 1076 genes with a significantly positive correlation with GMFG, which represented by dark red dots. Conversely, there were 1057 genes with obviously negative correlation with GMFG, which represented by dark green dots (FDR< 0.05). Two heatmaps shown respectively 50 significant genes positively and negatively related to GMFG (**Figures 11E, F**). And then, the intersection analysis between the differential genes of GMFG expression and the co-expressed genes from LinkedOmics database was carried out, and 71 genes were overlapping from the above analyses (**Figure 11C**). In order to make the correlation clearer, we drawn the correlation network diagram between GMFG and co-expressed genes using Cytoscape. As shown in **Figure 11G**, the red edges represented positive association with GMFG, and the blue edges represented negative association with GMFG. The width of the edge represented the strength of the correlation.

### Verification of the GMFG Expression in Breast Cancer Tissues by IHC

**Figure 12:**
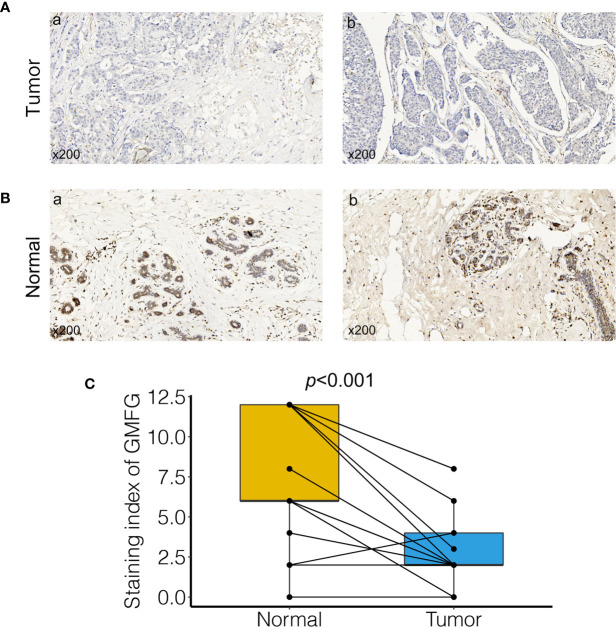
Validation of GMFG expression in breast cancer tissues by immunohistochemical (IHC). **(A)** GMFG expression in breast tumor (a: tumor tissue, staining intensity +, positive tumor cells 10%; b: tumor tissue, staining intensity +, positive tumor cells 25%). **(B)** GMFG expression in adjacent normal tissue (a: adjacent normal tissue, staining intensity +++, positive tumor cells 65%; b: adjacent normal tissue, staining intensity +++, positive tumor cells 70%). **(C)** Staining index of GMFG in nucleus in breast tumor and adjacent normal tissues.

To verify the results of bioinformatics analysis, IHC staining was performed on tissue microarray slides containing 24 paired breast tumor tissues and adjacent non-tumorous tissue samples. Characteristics and staining index of 24 patients were summarized in **Supplementary Table 1**. The level of the expression was quantitated by the staining index based on tumor cell proportion and staining intensity. The staining patterns of GMFG in tumor and normal tissues were shown in **Figure 12**, the expression level of GMFG was significantly down-regulated in breast tumor tissues compared with adjacent non-tumorous tissues (*p*<0.001), which was consistent with the results of bioinformatics analysis on RNA levels. Then, we analyzed the relationship between staining index of GMFG and clinicopathological characteristics. Although, there was no statistical correlation between the staining index of GMFG and clinicopathological characteristics (stage, T, N, ER status, PR status, HER2 status, grade and ki-67) in the results of IHC, the **Supplementary Figure 3** shown trends for GMFG protein expression to be higher in ER+ and HER2+ breast cancer. While not significant, the IHC study is underpowered and could yield a significant trend if more samples were assessed.

## Conclusion

Glia maturation factor γ (GMFG) is also known as the glia maturation factor beta homolog (GMFB-h) gene, which is a protein of 17-kDa. Studies have shown that the expression of GMFG gene was high in lung, thymus, spleen and colon, and the expression level of GMFG in human serum was different at various ages (16, 17). Previous research has shown that expression of GMFG was able to affect invasion and migration of ovarian cancer, and was correlate with survival rate of ovarian cancer (18, 19). Wang et al. also indicated that expression of GMFG was related to colorectal cancer metastasis (20). GMFG protein can regulate cytoskeleton reorganization of actin in microvascular endothelial and ovarian cancer cells, which was clearly an essential factor of many cellular processes including cytokinesis, endocytosis and chemotaxis, and affected the angiogenic sprouting in zebra fish (21–23). Moreover, GMFG can effected migration and adhesion of monocyte and regulate chemotaxis of neutrophil and T lymphocytes to participate in the immune response (24–26). However, the role of GMFG in the occurrence and progression of breast cancer and its potential function in tumor immunology is largely unknown.

In our study, we tried to determine genes related to immune that associated with the survival in BC patients from the TCGA database and GSE42568 dataset. We determined the genes correlated with ImmuneScore in BC samples from TCGA database using ESTIMATE algorithm and WGCNA analysis, and we verified the closely correlation between these genes and immunity through GO and KEGG enrichment analysis. Then we intersected immune-related genes with DEGs to obtain immune-related differential genes. Finally, survival analysis screened out the immune-related differential genes related to the prognosis of breast cancer, that is GMFG. Compared with normal breast tissues, the expression of GMFG was lower in BC tissues, which was further verified by IHC. Moreover, decreased expression of GMFG was significantly related to the poor prognosis. Besides, the expression of GMFG was related to the age, ER status, PR status, HER2 status and tumor size, which further suggested that the expression of GMFG was correlated with the subtype and the growth of tumor. The result of univariable and multivariable Cox regression analyses displayed that age, stage, the expression level of GMFG and radiotherapy were independent factor for predicting the prognosis of BC patients. Subsequently, a novel prognostic model for predicting the 3-year, 5-year and 10-year survival rate was developed based on the above four variables, and visualized as a nomogram. And the AUC values of the nomogram at 3-year, 5-year and 10-year were 0.897, 0.873 and 0.922, respectively. The prediction performance of the nomogram was better than that of the stage. In addition, we also found that GMFG expression level was correlative to sensitivity of some breast cancer chemotherapy drugs. Accordingly, GMFG has the potential to become a novel biomarker for the diagnosis and treatment of breast cancer.

A growing body of evidence suggests that the significance of TILs for predicting cancer progression has been revealed in multiple kinds solid tumor including breast cancer, which effected all phases of tumor growth (27, 28), as well as treatment response (29–31). Numerous studies have suggested that higher infiltration levels of TILs was related to improved survival and lower recurrences in breast cancer, especially in patients with HER2-positive and triple-negative breast cancer (32–34). However, different types of TILs may play contradictory roles in cancer. the recruitment of regulatory T cells (TR) might allow tumor cells to escape the immune response, which promoted the progression of invasive breast tumor and leaded to a significant shortening of OS in patients with invasive breast tumors. And we can observed that the level infiltration of TR was high in ER-negative tumors, high-grade tumors and patients with lymph node metastasis (9). In addition, Studies have shown that, during primary systemic chemotherapy (PSC), a low number of FOXP3-positive TR cell was related to favorable therapeutic outcomes in breast cancer (35). On the contrary, CD8+ lymphocytes were important component of cell-mediated immunity and tumor-infiltrating CD8+ lymphocytes have been proven to have anti-tumor activity in ovarian (36), colorectal (37) and esophageal tumors (38). About breast cancer, there were many research indicated that infiltrating level of CD8 + lymphocyte was obviously related to improve prognosis in patients with breast cancer (28, 39). According to reports, presence of CD8+ T cells in ER-negative breast tumors reduced the relative risk of death between 57% and 21%, while death risk in ER-positive and HER2-positive tumors were reduced by 27% (40). Moreover, CD8+ T lymphocytes were a crucial part of TILs related to response of chemotherapy in breast cancer (41). Therefore, the relationship between expression of GMFG with immunity was analyzed using GSEA. The results of GSEA indicated that signaling pathways associated with immune were significantly enriched in the GMFG high-expression group, such as T cell receptor signaling pathway, antigen processing and presentation and B cell receptor signaling pathway. Further CIBERSORT analysis suggested that multiple kinds T-cell was positively related to expression of GMFG in BC patients, such as CD8+ T cell, activated CD4+ memory T cells and gamma delta T cells. Importantly, CD8+ T cells have the strongest correlation with GMFG expression. A study showed that the GMFG was mainly located in pseudopodia of T lymphocytes,and the expression of GMFG was associated with T lymphocytes adhesion, cell migration, and chemotaxis (25). Therefore, the expression of GMFG was positively correlated with the infiltration amounts of CD8+ T-cell in breast cancer, which indicated that GMFG might play an antitumor role by increasing the infiltration of CD8+ T cells in breast cancer.

In summary, GMFG was a potential diagnosis and prognostic factor for BC patients. Decreased GMFG expression was correlated with poor prognosis, and the expression of GMFG was related to chemotherapy drugs sensitivity and clinical features including age, ER status, PR status, HER2 status and tumor size. Moreover, GMFG expression were negatively correlated with resting NK cells, macrophage M0 and macrophage M2. the immune infiltration levels in multiple T cell, especially CD8+ T cells, were positively related to the expression of GMFG. Therefore, GMFG might plays a vital antitumor effect in affecting the infiltration of immune cell, and could be used to predict the prognosis of breast cancer patients.

## Data Availability Statement

Publicly available datasets were analyzed in this study. This data can be found here: TCGA database:https://portal.gdc.cancer.gov/GSE42568: https://www.ncbi.nlm.nih.gov/geo/query/acc.cgi?acc=GSE42568 METABRIC database (https://ega-archive.org/access/data-access).

## Author Contributions

YY, XH, and Q-QT were involved in conception and design of the study. Y-FZ, S-RH, J-YZ, and H-FW were involved with revision of the article for important intellectual content. W-JS, P-JG, and Y-CS were involved with data interpretation. LW and J-WZ were involved in reading and approving the final version of the submitted manuscript as well as coordinating the entire process. All authors contributed to the article and approved the submitted version.

## Funding

Our research was supported by the National Natural Science Foundation of China (Grant No. 81772823).

## Conflict of Interest

The authors declare that the research was conducted in the absence of any commercial or financial relationships that could be construed as a potential conflict of interest.

## Publisher’s Note

All claims expressed in this article are solely those of the authors and do not necessarily represent those of their affiliated organizations, or those of the publisher, the editors and the reviewers. Any product that may be evaluated in this article, or claim that may be made by its manufacturer, is not guaranteed or endorsed by the publisher.

## Supplementary Material

Supplementary Figure 1 Biological functions of Immunescore-related genes. **(A)** The protein-protein interaction between the top 50 intra-module connectivity genes in yellow module. **(B)** KEGG pathway enrichment analysis for the top 50 intra-module connectivity genes. **(C–E)** GO have been analyzed and shows the Top 10 of biological process (BP), cellular component (CC) and molecular function (MF) for the top 50 intra-module connectivity genes.

Supplementary Figure 2 The profile and correlation analysis of TIICs in BC samples. **(A) **The bar plot display the proportion of 22 TIICs in breast cancer samples. **(B)** The heatmap display the relation between the proportion of 22 TIICs with expression of GMFG. The number in each small cell represented the correlation coefficient by Pearson coefficient test.

Supplementary Figure 3 Correlation analysis between the staining index of GMFG with clinical and pathological features in IHC data set.
