# Supplementary figures and images for: GMFG Has Potential to Be a Novel Prognostic Marker and Related to Immune Infiltrates in Breast Cancer
Source: Front Oncol. 2021 Jul 23;11:629633. doi: 10.3389/fonc.2021.629633 (PMC8343142; doi:10.3389/fonc.2021.629633)

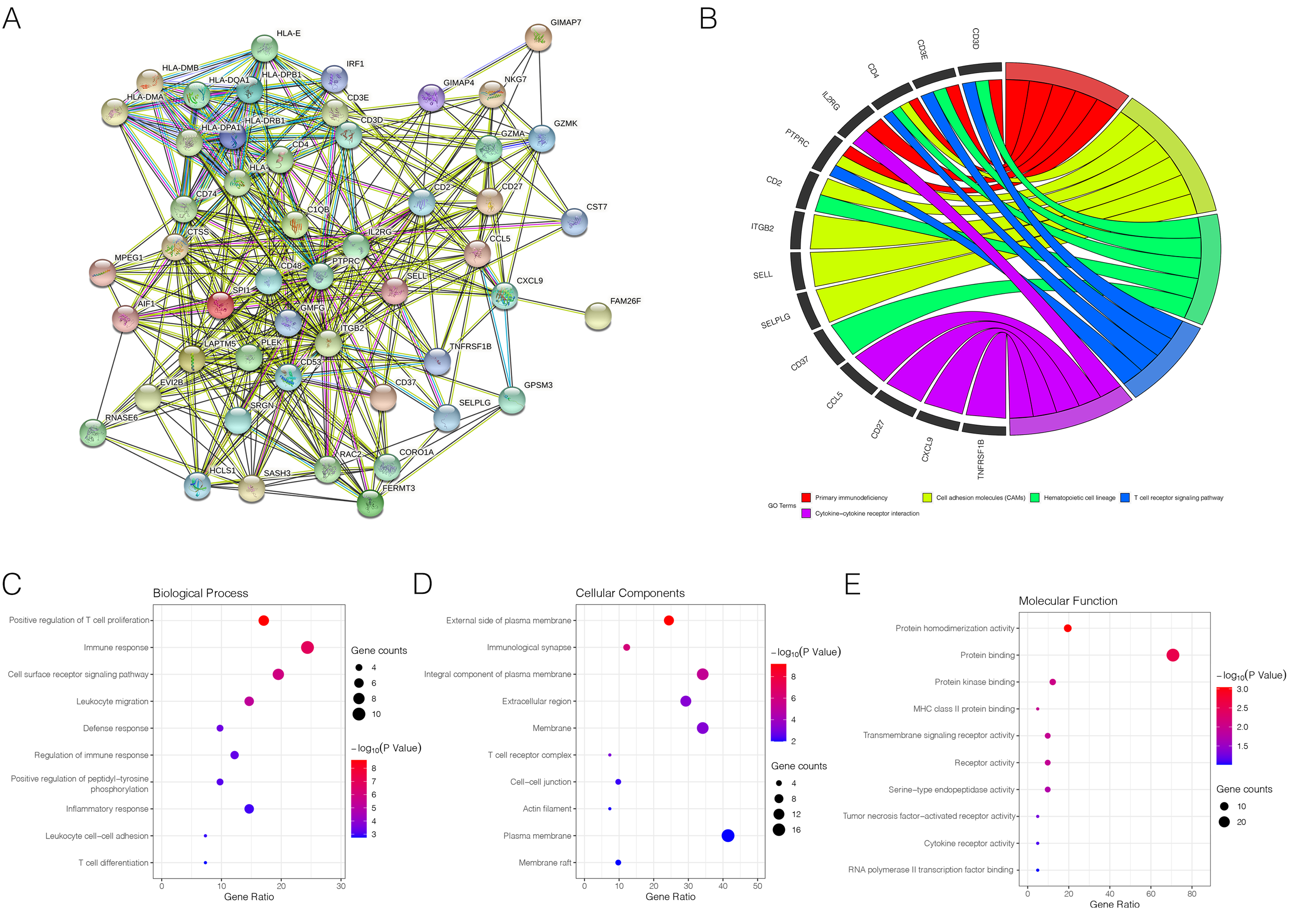

Supplement: Supplementary Figure 1 — Biological functions of Immunescore-related genes. (A) The protein-protein interaction between the top 50 intra-module connectivity genes in yellow module. (B) KEGG pathway enrichment analysis for the top 50 intra-module connectivity genes. (C–E) GO have been analyzed and shows the Top 10 of biological process (BP), cellular component (CC) and molecular function (MF) for the top 50 intra-module connectivity genes. [file Image_1.tif]

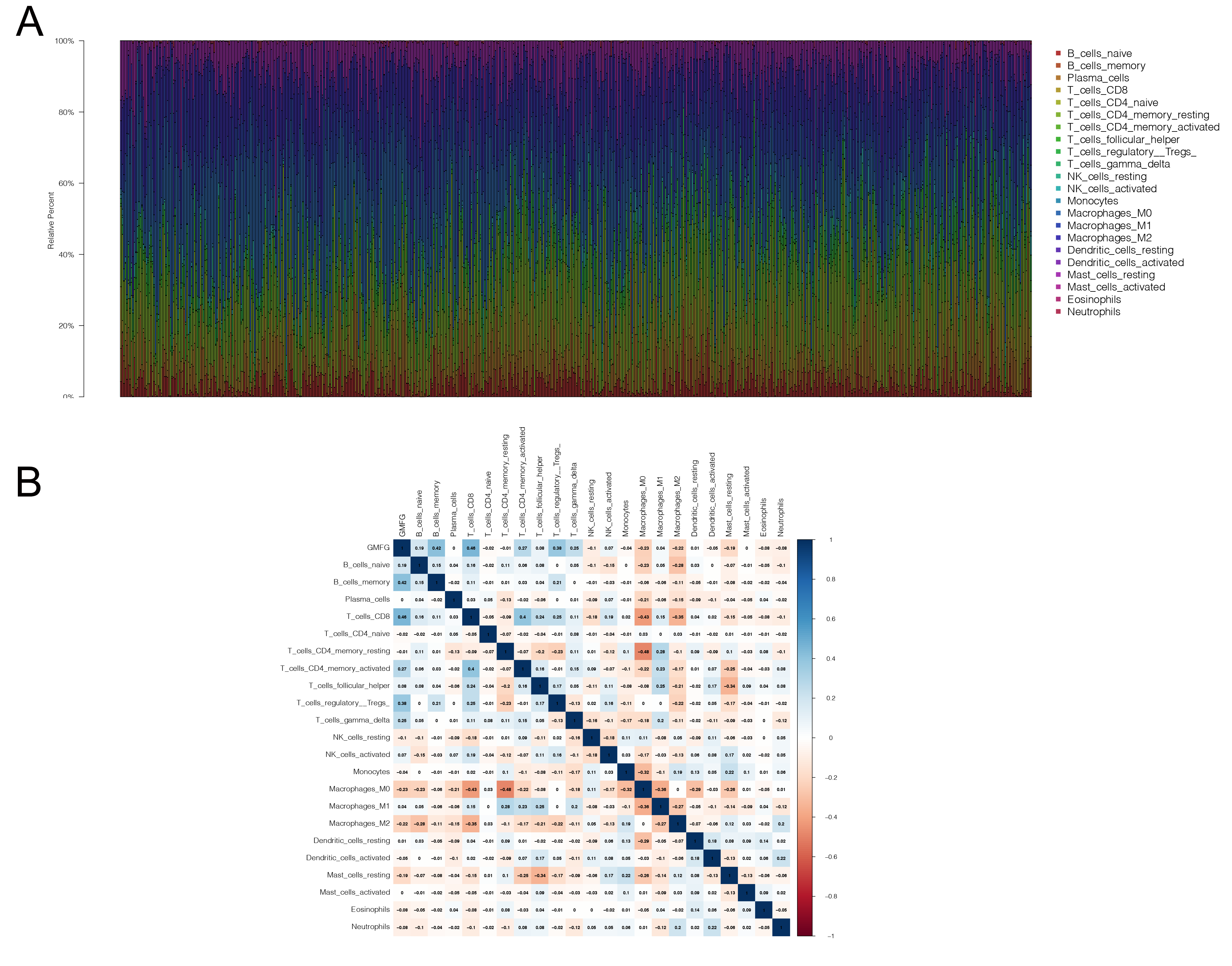

Supplement: Supplementary Figure 2 — The profile and correlation analysis of TIICs in BC samples. (A) The bar plot display the proportion of 22 TIICs in breast cancer samples. (B) The heatmap display the relation between the proportion of 22 TIICs with expression of GMFG. The number in each small cell represented the correlation coefficient by Pearson coefficient test. [file Image_2.tif]

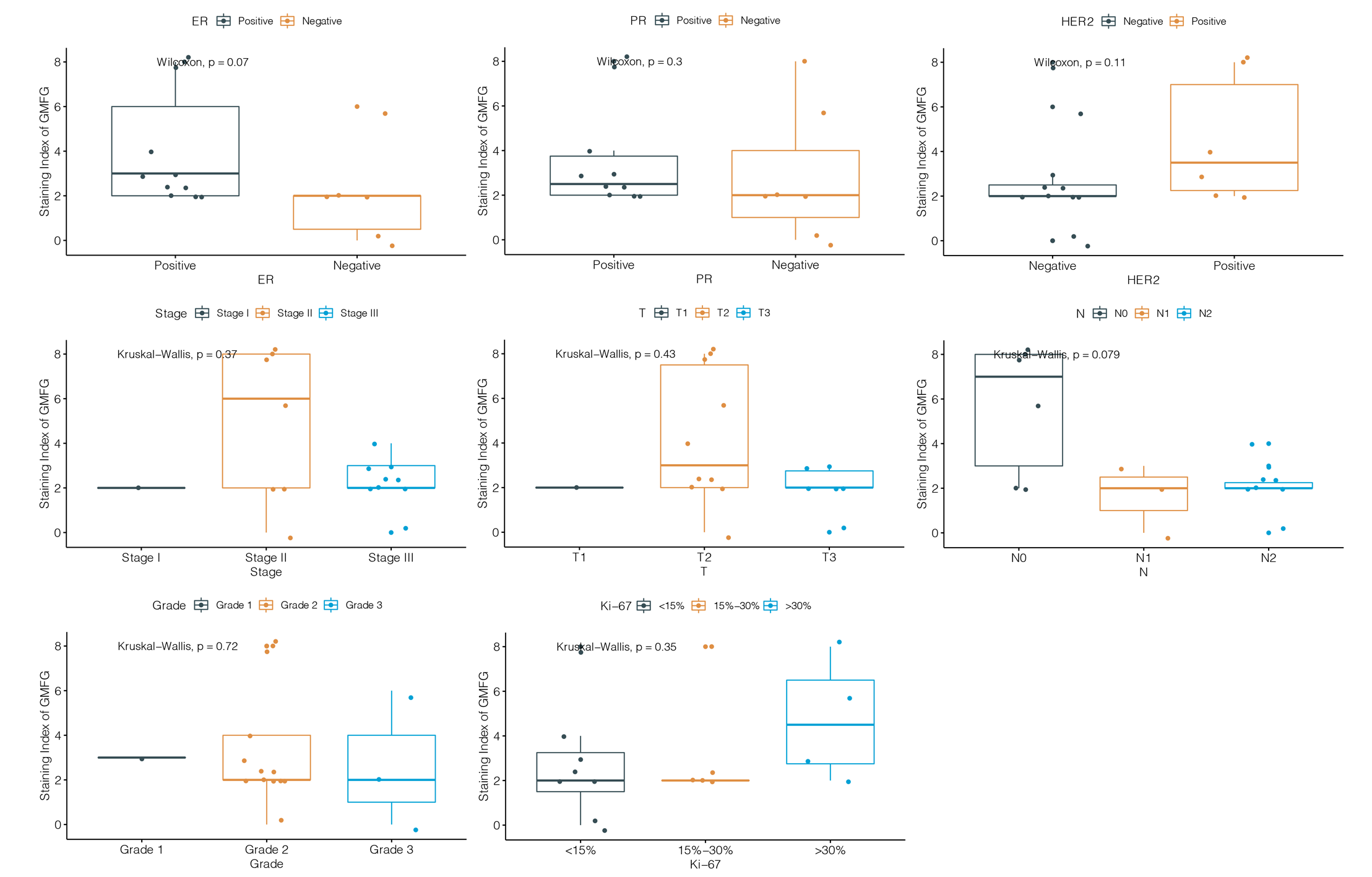

Supplement: Supplementary Figure 3 — Correlation analysis between the staining index of GMFG with clinical and pathological features in IHC data set. [file Image_3.tif]
