# Supplementary material for: GMFG Has Potential to Be a Novel Prognostic Marker and Related to Immune Infiltrates in Breast Cancer
Source: Front Oncol. 2021 Jul 23;11:629633. doi: 10.3389/fonc.2021.629633 (PMC8343142; doi:10.3389/fonc.2021.629633)
Supplement: Supplementary file 4 [file Table_1.docx]

Supplementary Table 1: Characteristics and staining index of 24 patients with breast cancer in IHC dataset.

| Sample | Age | Tissue | Grade | Stage | T | N | M | ER | PR | HER2 | Ki-67 | Staining Intensity | Tumor Cell Proportion | Staining Index |
| --- | --- | --- | --- | --- | --- | --- | --- | --- | --- | --- | --- | --- | --- | --- |
| A1 | 50 | Tumor | 1 | IIB | T2 | N1 | M0 | Positive | * | Negative | <15% | + | 10% | 2 |
| A2 | 50 | Normal | / | / | / | / | / | / | / | / | / | +++ | 65% | 12 |
| A3 | 50 | Tumor | 2 | IA | T1 | N0 | M0 | Positive | Positive | Negative | 15%-30% | + | 10% | 2 |
| A4 | 50 | Normal | / | / | / | / | / | / | / | / | / | * | * | * |
| A5 | 50 | Tumor | 1 | IIIA | T3 | N2 | M0 | Positive | Positive | Negative | <15% | + | 45% | 3 |
| A6 | 50 | Normal | / | / | / | / | / | / | / | / | / | * | * | * |
| A7 | 43 | Tumor | 2 | IIIA | T3 | N2 | M0 | Negative | Negative | Negative | <15% | - | - | 0 |
| A8 | 43 | Normal | / | / | / | / | / | / | / | / | / | ++ | 40% | 6 |
| B1 | 88 | Tumor | 2 | IIIA | T2 | N2 | M0 | Positive | * | Negative | 15%-30% | + | 10% | 2 |
| B2 | 88 | Normal | / | / | / | / | / | / | / | / | / | ++ | 30% | 6 |
| B3 | 56 | Tumor | 2 | IIIA | T2 | N2 | M0 | Positive | Positive | Positive | <15% | + | 55% | 4 |
| B4 | 56 | Normal | / | / | / | / | / | / | / | / | / | + | 10% | 2 |
| B5 | 47 | Tumor | 2 | IIA | T2 | N0 | M0 | Positive | Positive | Positive | >30% | ++ | 65% | 8 |
| B6 | 47 | Normal | / | / | / | / | / | / | / | / | / | +++ | 75% | 12 |
| B7 | 58 | Tumor | 2 | IIA | T2 | N0 | M0 | Positive | Positive | Negative | <15% | ++ | 70% | 8 |
| B8 | 58 | Normal | / | / | / | / | / | / | / | / | / | +++ | 60% | 12 |
| C1 | 84 | Tumor | 2 | IIIA | T2 | N2 | M0 | Positive | Positive | Negative | <15% | + | 25% | 2 |
| C2 | 84 | Normal | / | / | / | / | / | / | / | / | / | +++ | 20% | 6 |
| C3 | 54 | Tumor | 2 | IIB | T3 | N0 | M0 | Negative | Negative | Positive | 15%-30% | + | 10% | 2 |
| C4 | 54 | Normal | / | / | / | / | / | / | / | / | / | + | 20% | 2 |
| C5 | 35 | Tumor | 2 | IIIA | T2 | N2 | M0 | Positive | Positive | Negative | 15%-30% | + | 20% | 2 |
| C6 | 35 | Normal | / | / | / | / | / | / | / | / | / | ++ | 30% | 6 |
| C7 | 32 | Tumor | 2 | IIIA | T3 | N2 | M0 | Positive | * | Negative | * | + | 10% | 2 |
| C8 | 32 | Normal | / | / | / | / | / | / | / | / | / | ++ | 55% | 8 |
| D1 | 54 | Tumor | 2 | IIIA | T2 | N2 | M0 | * | * | * | * | - | - | 0 |
| D2 | 54 | Normal | / | / | / | / | / | / | / | / | / | ++ | 45% | 6 |
| D3 | 45 | Tumor | 2 | IIIA | T3 | N2 | M0 | Negative | Negative | Negative | <15% | + | 15% | 2 |
| D4 | 45 | Normal | / | / | / | / | / | / | / | / | / | ++ | 20% | 4 |
| D5 | 48 | Tumor | 2 | IIIA | T3 | N2 | M0 | Positive | Positive | Negative | <15% | + | 10% | 2 |
| D6 | 48 | Normal | / | / | / | / | / | / | / | / | / | ++ | 40% | 6 |
| D7 | 45 | Tumor | 2 | IIIA | T3 | N2 | M0 | * | * | * | * | - | - | 0 |
| D8 | 45 | Normal | / | / | / | / | / | / | / | / | / | * | * | * |
| E1 | 47 | Tumor | 2 | IIB | T2 | N1 | M0 | * | Positive | Negative | <15% | + | 10% | 2 |
| E2 | 47 | Normal | / | / | / | / | / | / | / | / | / | ++ | 60% | 8 |
| E3 | 42 | Tumor | 2 | IIIA | T3 | N1 | M0 | Positive | Positive | Positive | >30% | + | 45% | 3 |
| E4 | 42 | Normal | / | / | / | / | / | / | / | / | / | +++ | 75% | 12 |
| E5 | 49 | Tumor | 2 | IIB | T2 | N1 | M0 | Positive | Positive | Negative | >30% | + | 20% | 2 |
| E6 | 49 | Normal | / | / | / | / | / | / | / | / | / | +++ | 65% | 12 |
| E7 | 44 | Tumor | 2 | IIA | T2 | N0 | M0 | Positive | Negative | Positive | 15%-30% | ++ | 65% | 8 |
| E8 | 44 | Normal | / | / | / | / | / | / | / | / | / | +++ | 70% | 12 |
| F1 | 57 | Tumor | 3 | IIB | T2 | N1 | M0 | Negative | Negative | Negative | <15% | - | - | 0 |
| F2 | 57 | Normal | / | / | / | / | / | / | / | / | / | - | - | 0 |
| F3 | 43 | Tumor | 3 | IIA | T2 | N0 | M0 | * | * | Negative | <1% | ++ | 40% | 6 |
| F4 | 43 | Normal | / | / | / | / | / | / | / | / | / | +++ | 55% | 12 |
| F5 | 62 | Tumor | 3 | IIIA | T2 | N2 | M0 | Negative | Negative | Positive | 15%-30% | + | 10% | 2 |
| F6 | 62 | Normal | / | / | / | / | / | / | / | / | / | ++ | 15% | 4 |
| F7 | 40 | Tumor | 3 | IIA | T2 | N0 | M0 | Negative | Negative | Negative | >30% | ++ | 35% | 6 |
| F8 | 40 | Normal | / | / | / | / | / | / | / | / | / | +++ | 55% | 12 |
